# The Auditory-Visual Stroop Test to Assess Subjects with Tinnitus

**DOI:** 10.3390/brainsci16060565

**Published:** 2026-05-27

**Authors:** Anna Carolina Marques Perrella de Barros, Daniela Gil, Flavia Alencar de Barros, Richard S. Tyler, Ektor Tsuneo Onishi, Fátima Cristina Alves Branco-Barreiro

**Affiliations:** 1 Department of Speech-Language-Hearing Sciences, Escola Paulista de Medicina, Universidade Federal de São Paulo—UNIFESP, São Paulo 04023-062, Brazil; anna.perrella@unifesp.br (A.C.M.P.d.B.); dgil@unifesp.br (D.G.); branco.fatima@unifesp.br (F.C.A.B.-B.); 2 Tinnitus Clinic—Department of Otorhinolaryngology and Head and Neck Surgery, Escola Paulista de Medicina, Universidade Federal de São Paulo—UNIFESP, São Paulo 04023-062, Brazil; fab.suzuki@unifesp.br; 3 Department of Otolaryngology and Speech Pathology & Audiology, University of Iowa, Iowa City, IA 52242, USA; rich-tyler@uiowa.edu

**Keywords:** tinnitus, audiology, cognition, Stroop test, attention

## Abstract

**Highlights:**

**What are the main findings?**

- AV-Stroop assesses attentional and inhibitory control in tinnitus.
- Tinnitus shows broader executive control impairments.

**What are the implications of the main findings?**

- Tinnitus-matched sounds improve cognitive assessment sensitivity.
- Targeting attention/inhibition may improve tinnitus outcomes.

**Abstract:**

**Background/Objectives**: In this three-stage study, we aimed to adapt an Auditory-Visual Stroop test (AV-Stroop test) for tinnitus subjects, evaluate the correlation between performance in the conventional Stroop test (C-Stroop test) and the AV-Stroop test; assess the effect of cognitive screening test performance on the AV-Stroop test’s results; and apply the AV-Stroop test in participants with tinnitus and controls. **Methods**: At the First Stage, the AV-Stroop test was adapted using white noise (WN), pure tone (PT), and narrow band (NB) sound stimuli. At the Second Stage, results of the AV-Stroop test, the C-Stroop test, and the Montreal Cognitive Assessment (MOCA) were compared (*n* = 45). At the Third Stage, the AV-Stroop test was applied to participants with and without tinnitus (*n* = 70). The tinnitus group was assessed with an additional test track (stimuli matched to tinnitus spectral characteristics, Tinnitus Pitch). **Results**: We adapted 34 training and evaluation tracks for the AV-Stroop test. AV-Stroop test’s results were correlated with C-Stroop test’s total task time (WN, *p*-value = 0.002; NB and PT, *p*-value < 0.001 comparing C-Stroop word reading task; and WN, NB, and PT, *p*-value < 0.001 for C-Stroop color naming task), and number of errors (NB, *p*-value < 0.001 comparing C-Stroop word reading task, and *p*-value = 0.012 for C-Stroop color naming task). Participants’ MOCA scores were not associated with AV-Stroop test performance. Participants with tinnitus required more time and made more errors in the AV-Stroop test. Additionally, the tinnitus group made more errors in the Tinnitus Pitch track. **Conclusions**: The AV-Stroop test proved to be an accessible, easy-to-administer tool for evaluating attentional and inhibitory control in participants with tinnitus. The stimulus with spectral characteristics similar to tinnitus perception was more effective in assessing top-down executive control in participants with the symptom.

## 1. Introduction

The Stroop psychometric test [1] established the Stroop effect, based on conflicts between the dimensions of a stimulus, the expected dimension, and the dimension requested by the task. Automatic processing leads to errors in this task; conflict in the Stroop test demands controlled processing [2,3]. The conventional Color-Stroop (C-Stroop) paradigm is fundamentally based on word reading and color naming tasks. In this paradigm, cognitive conflict is established by presenting color-denoting words in incongruent colors, requiring the participant to inhibit the automatic reading response in favor of naming the color of the written word [1,3].

The Stroop effect is considered an ideal behavioral testing platform for evaluating the competition between stimulus-driven behavioral control and cognitive attentional control. The name Stroop has become synonymous with interference effects derived from incongruent stimuli that go beyond the verbal reactions and color stimuli originally reported [3]. Variations and adaptations of the conventional Stroop test have emerged over the years to ensure the conceptual replicability of the task and its applicability across different populations, including the tinnitus population.

Tinnitus is understood as a multisensory condition involving cognitive and emotional components [4]. This broader view of tinnitus has consequences for how we assess and manage the symptom. Tinnitus can affect cognitive task performance that demands executive attention control [5,6], including inhibition and cognitive flexibility [6]. Tinnitus can act as a distractor, increase response times and error rates, and reduce inhibitory control during interference tasks [5,6]. These findings emphasize the importance of specialized paradigms to detect these subtle impairments.

Research into the cognitive consequences of tinnitus consistently suggests a depletion of attentional resources. Tinnitus can be related to cognitive impairment extension—the greater the tinnitus severity, the greater the extent of cognitive deficits [7]. Furthermore, participants who do not seek specialized help and are supposedly habituated to the symptom can present diffuse concentration problems [8].

Previous investigations using behavioral paradigms have attempted to clarify executive dysfunction in the tinnitus population. As discussed in our recent scoping review [9], studies employing interference tasks have shown that tinnitus may interfere with top-down cognitive control [8,10,11,12,13,14,15,16].

Application of the Stroop test in tinnitus participants was discussed previously [9]. There are many reasons for the application of the Stroop paradigm in the tinnitus population. Stroop paradigms can help assess the executive function of tinnitus sufferers [8,10,11,12,13,14,15,17], highlight the neuronal connectivity through functional magnetic resonance imaging or electroencephalography assessments during an interference task [16,18,19], serve as an outcome measure pre- and post-treatment [20], and provide auditory-cognitive training [21,22,23].

The versions of the Stroop test used in the studies varied, encompassing different methodologies with visual, auditory, and emotional stimuli. When the stimuli involved the auditory modality, the effect of the spectral characteristics of the applied stimulus on participants’ performance was not explored. To the best of our knowledge, no previous study has used nonverbal stimuli and a stimulus compatible with the tinnitus-matched pitch for the application of the auditory modality of the Stroop test. In the Audiology field, tinnitus can be measured through psychoacoustic methods. Calibrated sounds are presented to the patient and matched to tinnitus using comparative methods.

The use of an acoustic stimulus with spectral characteristics similar to the auditory perception of tinnitus could help in a more precise evaluation of attentional and inhibitory control issues related to the symptom. The top-down evaluation can be more sensitive due to the stimulation of the specific bottom-up neuronal activity related to tinnitus. Therefore, this study aims to investigate failures in attentional and cognitive control mechanisms in subjects with tinnitus and to lay the foundation for future audiological interventions.

This study aims to demonstrate that interference paradigms using simple, nonverbal, topic-related stimuli can more effectively assess cognitive and attentional control in people with tinnitus, especially when the stimuli align with tinnitus perception, compared with Stroop protocols previously applied to the tinnitus population.

Building on the empirical foundation, in the present study, we aimed to study the following:

(1) Adapt the Auditory-Visual Stroop test (AV-Stroop test), an interference paradigm test, to assess executive control and attentional factors in participants with tinnitus (First Stage);
(2) Assess the correlation between performance in the conventional Stroop test (C-Stroop test) and the AV-Stroop test; evaluate whether participants’ scores on baseline cognitive screening tests are correlated with their results on the AV-Stroop task (Second Stage). In this stage, we predicted a significant positive correlation between AV-Stroop and C-Stroop test total task times and number of errors, while both measures were expected to remain independent of MOCA scores, indicating that the tasks capture specific executive attention rather than general cognitive decline;
(3) Apply the AV-Stroop test in participants with tinnitus and controls (Third Stage). It was hypothesized that the tinnitus group would exhibit significantly longer total task times and higher error rates than the control group, reflecting a deficit in inhibitory and attentional control. Finally, we expected a greater magnitude of interference (increased total task time and more errors) with the stimulus compatible with tinnitus perception.

## 2. Materials and Methods

**Figure 1:**
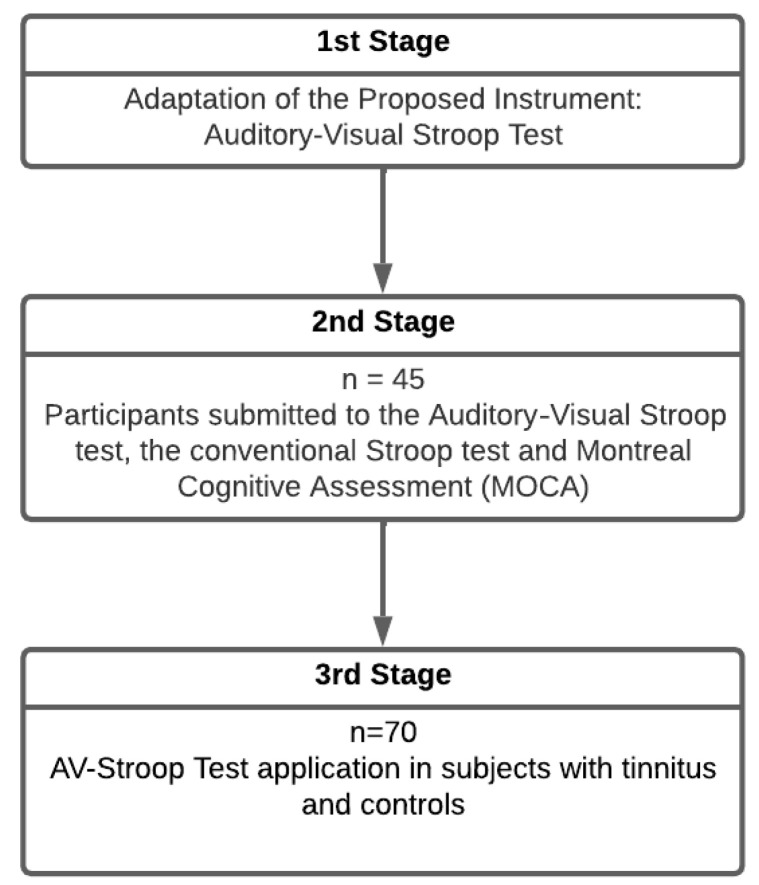
Flowchart with the study stages. Legend: MOCA, Montreal Cognitive Assessment; AV-Stroop Test, Auditory-Visual Stroop Test.

We utilized a three-stage study design: Stage 1 focused on the adaptation and technical preparation of the AV-Stroop test; Stages 2 and 3 employed a cross-sectional observational approach to compare results of the AV-Stroop test, C-Stroop test, and MOCA, and compare AV-Stroop test performance between groups with and without tinnitus (Figure 1).

This study was conducted at the Clinical Audiology Outpatient Clinic of the Department of Speech-Language-Hearing and Tinnitus Outpatient Clinic of the Department of Otorhinolaryngology and Head and Neck Surgery at the Federal University of São Paulo—Paulista School of Medicine (UNIFESP/EPM). The study was conducted in accordance with the Declaration of Helsinki, analyzed, and approved by the Research Ethics Committee (evaluation report 1133/2021). The participants voluntarily agreed to participate and signed an informed consent form.

Audiological measurements were performed in a standardized sound-treated booth. The AV-Stroop Test, C-Stroop Test, and MOCA were administered in a controlled, quiet clinical environment. Additionally, the auditory stimuli (used for the AV-Stroop test) were delivered via headphones. This setting was maintained to ensure consistency across all cognitive assessments and to provide a distraction-free space for participants. The acoustic conditions were monitored to ensure they remained stable and appropriate for the tests at a comfortable clinical level.

### 2.1. First Stage: Adaptation of the Auditory-Visual Stroop Test

The initial stage of this study was dedicated to the development and technical refinement of the AV-Stroop paradigm, with the development of training and test tracks. The experimental phase utilized three distinct test tracks—specifically the white noise (WN), narrow band (NB), and pure tone (PT) tracks. Each test track consists of the synchronized auditory and visual stimuli developed and standardized during the First Stage of this study.

No patient performance data were collected during this initial phase, as the primary objective of the First Stage was the technical adaptation and standardization of the AV-Stroop test. The results of this stage consisted of the finalized training and test tracks used in the subsequent experimental phases. The AV-Stroop test represents a novel adaptation of the classic Stroop paradigm [1], specifically designed to assess cognitive interference in the tinnitus population, using auditory and visual stimuli.

#### 2.1.1. Sound Stimuli Preparation

The AV-Stroop test used sound stimuli applied for pitch matching measurements: pure tone (PT), narrow band (NB), and white noise (WN). Frequencies included 0.25 to 8 kHz for PT and NB. Stimuli were recorded from the AC 40 audiometer (Interacoustics, Middelfart, Denmark) using an input cable to a digital recorder in WAV format, channel by channel (unilateral right, unilateral left, or binaural sound presentation), and then sent via a USB cable to the computer. The sound files were processed by Sound Forge Audio Studio 15. The output level for presentation in the test was calibrated to 0 dBFS (decibels relative to Full Scale) to ensure acoustic uniformity and eliminate intensity-related bias between different tracks. The acoustic presentation modes were set to right ear, left ear, or bilateral. Sound files were converted to MP3 320 kbps for greater fidelity.

#### 2.1.2. Equipment

The finalized digital test battery—consisting of the synchronized auditory and visual stimuli tracks for both training and experimental conditions—was compiled as computerized material ready for presentation via Microsoft PowerPoint 365^®^ (Forethought, Inc.; Microsoft Corporation; Sunnyvale, CA, USA), using a touchscreen notebook (11.6 inches, 1366 × 768 pixels, HD) and headphones (Koss Over-Ear UR22V-Koss Corporation; Milwaukee, WI, USA/China).

#### 2.1.3. Experiment Procedure

**Figure 2:**
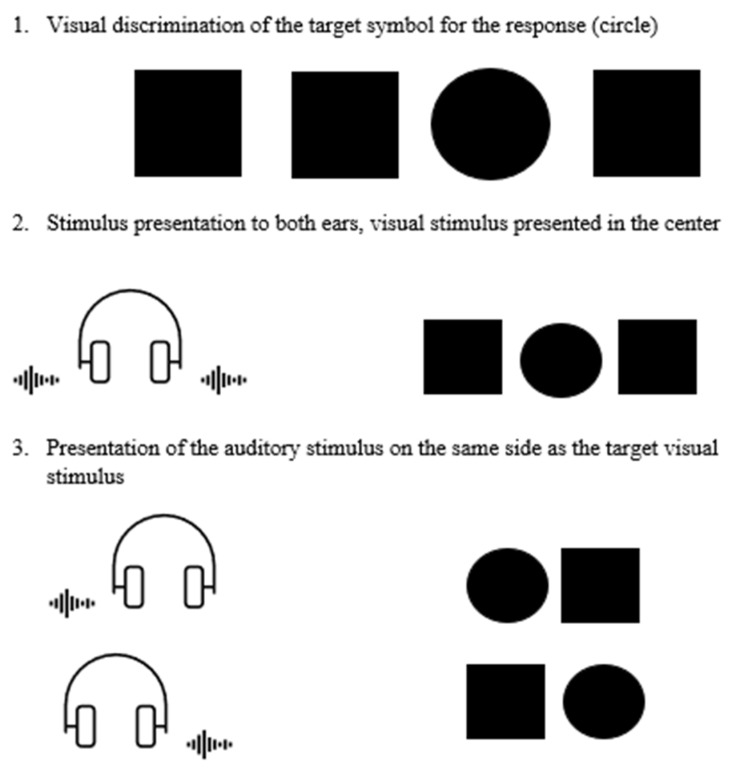
Training Stage: Visual symbol discrimination and auditory-visual association. Legend: Training track included congruent stimulation, with visual and auditory stimuli at the same side, to establish dominant form of presentation.

To establish the stimulus-response association, participants completed a pre-experimental training block (Figure 2). We designed the circle as the target visual stimulus for response, and the square(s) as distractor(s). Firstly, participants hear the recorded instruction to point to the circle to verify their visual discrimination of the target (the circle was presented among squares, no sound stimuli; Figure 2, Panel 1). Secondly, participants had an instruction trial, where they hear the recorded instruction “When you hear the sound, point to the circle” (in the following trials, sound stimuli were presented before the visual stimuli; Figure 2, Panels 2 and 3).

During the training block, participants were familiarized and habituated to the rule that auditory stimulation (sound source) dictated the correct response side. Immediate feedback was provided during training to ensure the association was correctly internalized. In the training conditions illustrated in Figure 2 (Panels 1 and 2), each stimulus was presented once to the participant to ensure clear identification of the target before proceeding to the continuous trials of congruent stimulation.

At the beginning of the training track, the sound stimulus was presented binaurally for a single trial to verify stimulus recognition and calibrate the participant’s centered auditory perception (target visual stimulus presented in the center of two distracting stimuli; Figure 2, Panel 2). This was followed by the alternated right and left presentation to establish the spatial-auditory association (target visual and sound stimuli on the same side; Figure 2, Panel 3), completing 18 sequential trials with congruent presentations (9 on the right side, 9 on the left side). This amount was established to facilitate rapid familiarization and habituation, and ensure participants reached a stable performance baseline before the start of the experimental stages. Although the specific spatial mapping (left ear/left circle, right ear/right circle, or binaural/center) was not explicitly stated in the verbal instructions of the training block, the congruent spatial orientation served as an intuitive mapping. This stage allowed for the implicit association between the auditory stimuli and the visual target. By establishing this clear link during the training phase, we ensured that participants had a stable procedural baseline before the experimental tracks introduced cognitive interference. Additionally, that AV-Stroop test task execution relied on rapid cognitive interference processing rather than conscious rule-deduction.

**Figure 3:**
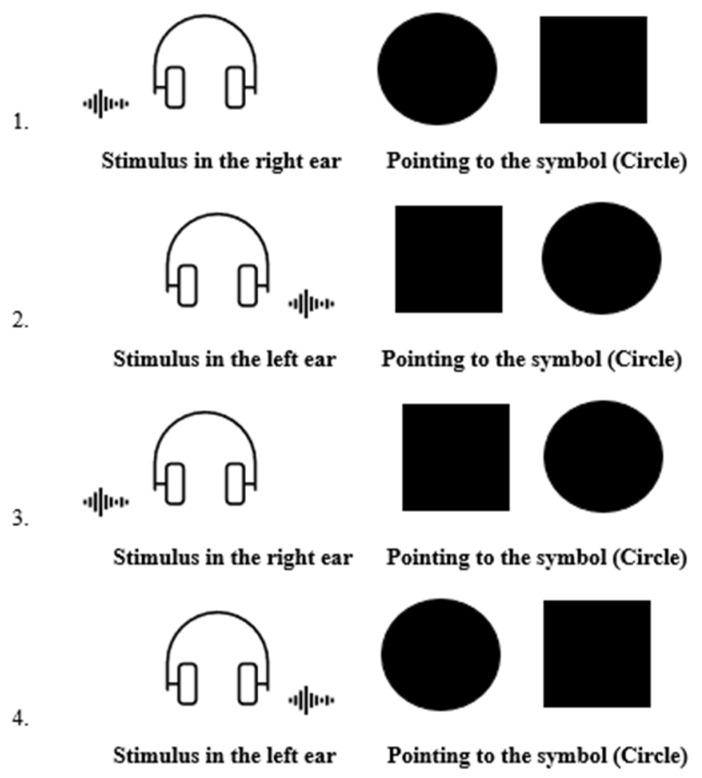
Test presentation scheme. Legend: (**1**) congruent situation, with visual and auditory stimuli on the right side; (**2**) congruent situation, with visual and auditory stimuli on the left side; (**3**) incongruent situation, with visual stimulus on the left side and auditory stimulus on the right side; and (**4**) incongruent situation, with visual stimulus on the right side and auditory stimulus on the left side.

Following the training block, the experimental test trials were administered immediately without delay to ensure the retention and strength of the stimulus-response association. After the recorded instruction “When you hear the sound, point to the circle as fast as you can and without making mistakes”, four stimulus presentation conditions were developed for the test tracks, including congruent and incongruent trials (Figure 3). The participant’s task was to point the circle at the screen as quickly as possible and avoid errors. AV-Stroop test tracks had 60 trials of stimuli, randomly distributed according to the condition: 80% of the presentations were in congruent situations (48 stimulus sequences, 24 per ear, auditory stimulus and visual target on the same side), and 20% of the presentations were in incongruent situations (12 sequences, six per ear, auditory stimulus and visual target on opposite sides). The ratio of congruent/incongruent stimuli was manipulated to create a rare stimulus effect for the incongruent trials. Each test track was about 120 s, and each sound stimulus had 5 s. By the time the participant touches the visual target, the trials of experimental stimuli are subsequently presented. The target circle and distractor squares were approximately 4 cm in size, with a lateral displacement of 5 cm from the central fixation point. Stimuli were displayed on the same visual display monitor for all participants to ensure consistent stimulus size, brightness, and response latency across the study.

AV-Stroop test tracks followed sequences and organizations’ patterns (Table 1).

Visual stimuli were programmed to appear after sound stimuli, delayed by 0.5 s. This delay ensured that the auditory stimulus was fully registered, familiarizing and habituating the participant to focus on the acoustic track before the visual component triggered the cognitive interference task. The sound stimulus was the same during each training or test track. The total task time was recorded automatically. The total task time was defined as the total it took to run the whole test. Errors (pointing to the distracting visual stimulus/double-clicking on the screen) were manually counted.

#### 2.1.4. Protocol Observational Validation

The evaluation of the First Stage was primarily observational and focused on procedural feasibility. The instrument underwent an internal pilot trial (*n* = 5) to refine the stimulus parameters. During this phase, the initial pool of tracks was screened for acoustic uniformity and perceptual clarity, resulting in the selection of the final 34 test tracks, each one composed of 60 trials of stimuli. The final tracks consisted of three distinct types of auditory stimuli: PT, NB, and WN. For the PT and NB conditions, the stimuli covered frequencies ranging from 0.25 to 8 kHz. This range was selected to encompass the standard clinical frequencies assessed in diagnostic audiometry, thereby evaluating attentional and inhibitory control across the spectrum most relevant to human hearing and clinical evaluation.

The number of training trials was determined through the internal pilot study, which indicated that a 21-series sequence was sufficient for participants to achieve procedural fluency and full task comprehension. Throughout the main study, this training duration proved effective, as participants consistently demonstrated the ability to perform the task without further clarification. The training track with 18 trials of stimuli was sufficient to establish the spatial mapping and achieve stable total track times, while maintaining a brief test duration to prevent participant fatigue.

During the internal pilot trial, data were collected on participant response accuracy and verbal feedback. Observations confirmed that the 21-series training track was sufficient for 100% of participants to achieve stable task comprehension and stimulus discrimination. Consequently, no further modifications were required before the main experimental phase.

#### 2.1.5. Technical Specification of the AV-Stroop Test

The structure of the AV-Stroop test used in this study consisted of three main components designed to evaluate interference in cognitive control:

- Auditory Stimuli: The test utilizes clinical pitch-matching stimuli consisting of pure tone (PT), narrow band (NB), and white noise (WN) tracks across standard frequencies (0.25 to 8 kHz).
- Visual Stimuli: The visual stimuli were delivered via digital presentation software (Microsoft PowerPoint) on a standard monitor. The display featured a consistent visual field where circle and square markers were positioned in the left and right hemifields of the screen. The circle served as the target for response detection, and its spatial location was synchronized with the corresponding auditory channels.
- Congruent and Incongruent Conditions:

* Congruent: The auditory stimulus and the visual target are aligned (e.g., sound in the left ear corresponds to the visual target on the left).
* Incongruent: The auditory stimulus and visual target are misaligned (e.g., sound in the left ear corresponds to the visual target on the right).

This setup creates a cross-modal interference task where the participant must resolve the spatial conflict between the two sensory inputs, a process requiring inhibitory control rather than simple spatial orientation.

- Outcome Measures: The primary parameters recorded are total task time and number of errors.

Table 2 summarizes the main characteristics of the training and the test tracks.

This identical structural blueprint was strictly maintained throughout the experimental applications in Stages 2 and 3 of the study.

### 2.2. Second Stage: Comparison of the Auditory-Visual Stroop Test, the Conventional Stroop Test, and the Montreal Cognitive Assessment Results

The Second Stage was the primary experimental trial, and involved 45 volunteers (28 females and 17 males), aged 20 to 57 years (mean 39.6 years, standard deviation 13.39 years), selected considering the eligibility criteria (Table 3). At this stage, participants were included regardless of the presence or absence of tinnitus to establish the comparison between the in-person assessments. By comparing an individual’s performance across the experimental conditions, any potential baseline cognitive influence related to the presence of tinnitus was held constant, ensuring that the results specifically reflect the comparison of the participant’s performance across the conditions.

The subsequent testing utilized the AV-Stroop test adapted during the First Stage of this study. The auditory stimuli consisted of three selected tracks utilizing 1 kHz PT, 1 kHz NB, and WN (1 kHz is a central frequency, and WN provides a wide range of stimulation at different frequencies). The task began with the training track to establish the conditioning before proceeding to the test tracks.

The C-Stroop test was based on word reading (WR, participants read the names of colors) and color naming (CN, participants named the colors of the written names—color names) tasks [25]. Pretest included a check of color recognition, reading training, and color naming (words “pink/black/green/blue” written in different colors). For the evaluation stages, a card with 112 words written sequentially in four columns was used. The names of the four colors were written in different colors and randomly distributed. First, the participant was asked to read the words, in column order, as quickly as possible. Later, the participant was instructed to name the colors, in column order, as quickly as possible. The C-Stroop test’s total task time and number of errors were manually registered by researchers.

The MOCA test [26,27] assessed eight cognitive domains: visuospatial function and cognitive function; naming; immediate memory with five-word repetition; attention with direct and indirect digit span; language and verbal fluency; abstraction; delayed recall; and spatial and temporal orientation. The total test score was considered for the analysis.

Assessments followed a fixed sequence (MOCA, C-Stroop test, AV-Stroop test). The brief duration of the battery facilitated high participant engagement and prevented reported fatigue, ensuring that total task times reflected performance rather than cognitive exhaustion.

### 2.3. Third Stage: Application of the Auditory-Visual Stroop Test in Participants with Tinnitus and Controls

The Third Stage, a sequential experiment, utilized an extension of the Second Stage cohort (*n* = 45), with the inclusion of 25 additional participants. The final sample for this stage totaled 70 participants. The eligibility criteria remained the same (Table 3). Additionally, the Study Group (*n* = 30) included participants with idiopathic tinnitus, constant subjective, unilateral or bilateral, and whistling or hissing type. The Control Group (*n* = 40) was composed of participants without tinnitus. All participants from Stage 2 met these specific criteria and were transitioned into the Stage 3 analysis, supplemented by 25 newly recruited participants. Groups had no difference regarding age, gender, hearing level, educational level, anxiety, and depression level (Table 4).

Participants in the Control Group provided demographic and identification data, including age and gender, to ensure an accurate characterization of the study population. Additionally, they provided general health information to identify relevant comorbidities. Afterwards, the Hospital Anxiety and Depression Scale (HADS) [28,29] was applied. Participants in the Study Group underwent a structured anamnesis and clinical intake to record tinnitus-specific data (onset, duration, and type of perception) and relevant health comorbidities. This was followed by the HADS, the Tinnitus Handicap Inventory (THI), and pitch and loudness matching. The characteristics of the Study Group are shown in Table 5.

The HADS was applied to verify the homogeneity of the sample, considering symptoms of anxiety and depression. The analysis was based on the absolute total scores. Qualitative classifications of the results were excluded to maintain a quantitative assessment of the participants’ performance. The Tinnitus Handicap Inventory (THI) [30,31] classified tinnitus severity in the Study Group according to its impact on quality of life [32].

Each participant in the Study Group was submitted to the tinnitus pitch and loudness matching to define tinnitus perception in terms of frequency (pitch) and intensity (loudness) [33]. The procedure was performed in an acoustic booth using an audiometer (Interacoustics AD 229). To determine the stimulus type that most closely resembled the participant’s tinnitus, we presented pure tone (PT), narrow band (NB), and white noise (WN). The presentation mode was ipsilateral (sound stimuli were presented in the same ear as the tinnitus perception). The PT and NB stimuli were reproduced at frequencies from 0.25 to 8 kHz. The presentation was performed at 20 dB SL (sensation level) per frequency for 5 s. Stimuli were subjectively compared by the participants to identify the perceptual likeness of the sound in relation to their tinnitus. This measurement allowed the definition of the type and frequency of tinnitus sensation (pitch) [17]. The tinnitus pitch-matching procedure was retested and confirmed. This approach ensured that the Tinnitus Pitch track reflected a stable subjective percept, thereby enhancing the reliability of the custom-tailored auditory stimuli.

To investigate the sensation of intensity, the tinnitus pitch stimulus defined for each Study Group participant was presented 5 dBHL (hearing level) below the auditory threshold, with increments of 5 dBHL at each level for 5 s, until the participant reported the level at which the presented stimulus resembled their tinnitus [33]. The tinnitus loudness-matching procedure was retested and confirmed.

All participants performed the training track. The AV-Stroop test was applied in three pattern tracks (1 kHz PT, 1 kHz NB, and WN). The training and the test tracks followed the identical format and trial structure established in the First Stage. For tinnitus participants, there was an additional test track with a stimulus compatible with the type of tinnitus and the frequency recognized in the pitch measurement (Tinnitus Pitch). AV-Stroop tracks were administered in a fixed order (WN, NB, PT, Tinnitus Pitch). The task’s brevity and simplicity ensured high participant engagement and precluded reported fatigue, allowing performance to be attributed to stimulus-specific interference. The participants’ performance was analyzed by the total task time (seconds) and the number of errors.

### 2.4. Statistical Analysis

Data were first characterized through descriptive statistics, including summary measures such as mean, median, minimum and maximum values, standard deviation, and absolute and relative frequencies (percentages).

Inferential analyses were conducted to compare groups and evaluate the associations between the experimental variables. Comparing participants with and without tinnitus, the Mann–Whitney test was employed for continuous variables, and the Chi-Square test of Homogeneity was used for categorical data. Within the tinnitus group, the Friedman test was applied to compare variables across different test tracks.

Effect sizes for paired non-parametric comparisons were calculated using the rank-biserial correlation, while the magnitude of association across multiple conditions was assessed using Kendall’s coefficient of concordance.

To assess the relationship between total task times and the number of errors (across C-Stroop, MOCA, and AV-Stroop tests), Spearman’s rank correlation coefficients were calculated. For each coefficient, the 95% confidence interval and the *p*-value for the significance of the correlation (testing the null hypothesis of no association) were reported. For all analyses, the significance level was set at 5%.

## 3. Results

### 3.1. First Stage: Adaptation of the Auditory-Visual Stroop Test

The initial phase resulted in 34 standardized test tracks specifically adapted for the tinnitus population. The resulting stimulus set consisted of auditory signals paired with visual targets and distractors. Training and test tracks were created on the computer in three stimulus categories (WN, NB, and PT), with the same formatting, totaling 2 WN tracks, 16 NB tracks, and 16 PT tracks.

Training tracks had 21 presentation trials of stimuli, the first slide with the instruction to “point to the ball”, and the presentation of the target visual symbol among distracting elements. Next was the slide with the test instruction, “When you hear the sound, point to the circle as fast as you can and without making mistakes”, accompanied by the on-screen instruction “click here to continue”, which the participant touched to transition to other sequences, with congruent presentations of the auditory and visual stimuli.

AV-Stroop test assessment tracks had 61 presentation trials of stimuli, the first slide with the test instruction, “When you hear the sound, point to the circle as fast as you can and without making mistakes”, accompanied by the on-screen instruction, “Click here to continue”. The participant touched the screen and transitioned to other sequences, with congruent and incongruent auditory and visual stimuli presentations.

Each track was successfully validated for clinical application and stimulus-response consistency. This stage concluded with the establishment of a finalized experimental protocol, which served as the standardized framework for the subsequent data collection phases.

The validation of the test tracks was concluded after the pilot phase (*n* = 5) demonstrated that the stimulus parameters met the required functional criteria. Specifically, validation was defined by 100% participant accuracy in target identification and unanimous procedural habituation and familiarization with the AV-Stroop tasks. These results confirmed that the 21-series training sequence effectively established the necessary procedural baseline, allowing the finalized tracks to be implemented in the subsequent experimental stages.

### 3.2. Second Stage: Comparison of the Auditory-Visual Stroop Test, the Conventional Stroop Test, and the Montreal Cognitive Assessment Results

Although the inclusion criteria excluded evident and/or diagnosed neurological disorders, MOCA total scores ranged from 15 to 29 (Mean = 24.067; SD = 3.360). These results demonstrated significant cognitive variability within the sample in the Second Stage. MOCA is a tool with a total score of 30 points. A score of 26 or higher is established as the normative threshold for cognitive health, while scores below this point are indicative of potential mild cognitive impairment (MCI), according to the original validation [26].

AV-Stroop test (WN, NB, and PT) total task time was positively associated with the total task time in WR (WN, *p*-value = 0.002; NB, and PT, *p*-value < 0.001) and CN stages of the C-Stroop test (WN, NB, and PT < 0.001) and not associated with MOCA score (WN, *p*-value = 0.225; NB, *p*-value = 0.136; and PT, *p*-value = 0.185) (Table 6).

The AV-Stroop test’s number of errors (WN, NB, and PT) was positively associated with the C-Stroop test in the WR stage (WN, *p*-value = 0.006; NB, *p*-value < 0.001, and PT, *p*-value = 0.008), with the C-Stroop test number of errors in the CN stage (NB, *p*-value = 0.012), and not associated with the MOCA score (WN, *p*-value = 0.144; NB, *p*-value = 0.254; and PT, *p*-value = 0.454) (Table 7).

Table 6 and Table 7 present both significant and non-significant associations. While several correlations did not reach statistical significance, the 95% Confidence Intervals were included to illustrate the direction and precision of the estimated effects. The moderate strength correlations observed demonstrated agreement between the AV-Stroop and C-Stroop tests. Furthermore, scores from the cognitive impairment screening instrument did not appear to modulate performance on the proposed test, suggesting that the AV-Stroop test specifically targets executive attention mechanisms.

### 3.3. Third Stage: Application of the Auditory-Visual Stroop Test in Participants with Tinnitus and Controls

Evidence indicated a significant difference between the Study and Control groups in the total task time distributions for both training and test tracks (training track, *p*-value = 0.008; WN, *p*-value = 0.018; NB, *p*-value = 0.006; and PT, *p*-value < 0.001) (Table 8). All conditions of the AV-Stroop test (training and test tracks) showed consistent moderate effects ranging from 0.33 to 0.49. This confirms that tinnitus participants had significantly and meaningfully altered total task times compared to controls.

Table 9 presents the descriptive analysis of the number of errors for each group on each test track.

Errors were analyzed by the distribution of participants of the Study and Control Groups. Participants were categorized into two performance tiers based on an error threshold: those with <3 errors and those with ≥3 errors. The comparative analysis of participant error-rate frequencies revealed a significant difference in the distribution of the Study and Control groups for the WN and NB tests (*p* = 0.045 and *p* = 0.012, respectively). Specifically, a higher proportion of participants in the Study Group fell into the high error category (≥3 errors). For the PT test, the difference in participant distribution between groups did not reach statistical significance (*p* = 0.079). The Study Group showed a higher concentration of participants with three or more errors in the AV-Stroop test tracks. These findings are summarized in Table 10.

The results comparing the WN, NB, and PT test tracks with the Tinnitus Pitch track performed in the Study Group showed no significant differences in total task time (*p*-value = 0.907). The analysis conducted within the Study Group compared the impact of the different acoustic stimuli on performance accuracy. The analysis used the test track type (WN, NB, PT, and Tinnitus Pitch) as the independent variable and the total task time, and the number of errors as the dependent variable. A significant difference in error rates was found between the four tracks (*p*-value = 0.020), indicating that the type of acoustic interference significantly influenced the participants’ task accuracy (Table 11).

While the error comparison between WN and Tinnitus Pitch yielded a marginal trend (*p* = 0.059), the overall pattern of results—including significant differences in PT and Tinnitus Pitch, and narrow band and Tinnitus Pitch comparison (*p*-value = 0.003, and *p*-value = 0.046, respectively)—suggests that the personalized track induces a higher cognitive load (Table 12). Analysis of the error distributions yielded small effect sizes (Kendall’s W = 0.07 to 0.15). Although effect sizes were small, the tinnitus pitch-matched condition demonstrated a consistent pattern of increased interference. The low error rates across conditions suggest that participants performed the task with high accuracy, supporting the use of total task time as a more sensitive indicator of cognitive interference.

## 4. Discussion

### 4.1. First Stage: Adaptation of the Auditory-Visual Stroop Test

The AV-Stroop training and test tracks were developed using different sound stimulus types with different spectral characteristics. Tinnitus mechanisms can involve tonotopic modifications at the central level [34]. Physical properties of the stimulus can activate different auditory fibers, given auditory pathway tonotopy.

We hypothesized that spectral characteristics of the stimulus could interfere with the AV-Stroop test’s performance. In addition, stimuli with spectral characteristics compatible with tinnitus auditory perception can be more effective in evaluating attentional issues and inhibitory control in tinnitus participants. These stimuli can evoke even more specific neuronal activity related to tinnitus.

Stroop’s tasks can involve activations in areas such as the lateral prefrontal cortex, posterior parietal cortex, occipitotemporal cortex, medial prefrontal cortex, anterior cingulate cortex, and interactions with the cerebellum [35]. The anterior insula, anterior and posterior cingulate cortex, precuneus, medial and dorsolateral prefrontal cortex, and inferior parietal lobule are areas related with suffering, embodiment, functional, and social impact [36].

Some studies aimed to increase the sensitivity of the Stroop test for application in tinnitus participants [10,11,12,16,18,19]; for a review, see [9]. AV-Stroop test adaptation resembles another study. The auditory stimuli were presented on the right and left sides. Additionally, the association with the presentation side establishes a conflict between the stimulus dimensions [16].

Stimuli presented only in congruent conditions (training stage) familiarized and habituated the participant to the most expected form of presentation—auditory and visual stimuli on the same side. Logical association was determined by the presentation way of the stimuli in sensory modality: “The side I hear is the side I see”. Hence, the interference effect can be more effective, instinctive, and basal in test tracks.

The adequacy of the training phase was confirmed by the performance data collected during the protocol observational validation (*n* = 5), where participants achieved an accuracy rate of 100% and demonstrated stable total task times by the conclusion of the 21-trial sequence. This objective evidence supports the conclusion that the training track was sufficient for procedural mastery and full comprehension of the task demands, even without reaching the threshold of behavioral automation.

We manipulated the distribution of congruent and incongruent trials for the test tracks. Incongruent trials accounted for 20% of presentations (rare stimulus effect), requiring attentional resources to inhibit the automatic response. Another study manipulated and reduced the proportional presentation of emotionally relevant words to increase the interference effect in the emotional Stroop [12]. Other methodology presented more balanced distributions of congruent and incongruent trials [16] or a higher percentage of incongruent stimuli [17].

We used nonverbal auditory stimuli, unlike previous studies that applied Stroop in the auditory modality [16,21,22,23]. We sought to establish a simplified process for the dominant association and for the conflict. There was no bias towards the concept of laterality or aspects of semantics or language. Even the visual stimuli we used were basic, graphic, familiar geometric shapes that were easily differentiated.

We determined the total task time as a quantitative variable, as previously done [10,11]. Some studies established reaction time [8,12,13,14,15,16,17,18,19,20], with specific results for congruent and incongruent presentation trials, and a more detailed analysis of the interference effect.

### 4.2. Second Stage: Comparison of the Auditory-Visual Stroop Test, the Conventional Stroop Test, and the Montreal Cognitive Assessment Results

During the Second Stage, the number of registered errors was analyzed—studies’ second most analyzed variable [8,15,20]. Some studies used accuracy as an outcome measure [16,17]. Two studies mentioned recorded errors [8] or correct answers [16], whereas other studies did not report this information explicitly [15,17,20]. Desired functionalities created for training and test tracks—instructions, on-screen guidance, response identification, and path to presentation trials—had the expected effect.

The AV-Stroop test results were compared with the reference conventional test version in the same sample (Table 6 and Table 7). Stroop tasks in auditory and visual modalities may be conceptually equivalent, considering the correlation between participants’ individual scores in the two tasks [37]. The moderate correlation observed between the two instruments supports their convergent validity in measuring attentional and inhibitory control (Table 6 and Table 7). However, the AV-Stroop captures a distinct dimension of executive function, as it extends to the auditory domain. Our proposal consisted of a conceptual adaptation, like previous studies [8,13,14,15,16,17,19,20].

C-Stroop test’s modifications could have a discrete effect on its magnitude but not on its quality. Our results reinforce a generalization effect of the desired interference phenomenon [38]. The number of errors in the AV-Stroop test was positively correlated with the Stroop CN task only for the NB, showing greater efficiency of this stimulus for assessment (Table 7).

The MOCA score did not interfere with the AV-Stroop test (Table 6 and Table 7), reinforcing that our proposal involved a simple, basal, easy-to-apply task. MOCA scores exhibited a wide distribution, indicating varied global cognitive profiles despite the absence of formal neurological diagnoses. The null association between MOCA scores and AV-Stroop performance suggests that the attentional and inhibitory control required for the AV Stroop tasks may be a distinct executive domain, independent of general cognitive screening metrics.

### 4.3. Third Stage: Application of the Auditory-Visual Stroop Test in Participants with Tinnitus and Controls

Tinnitus can have a generalized effect on cognitive performance. The tinnitus group required more time to complete all Stroop measures, even in congruent conditions (training track) (Table 8). The generalized difference involved tasks that required controlled processing and those that required automatic processing. Other studies demonstrated longer reaction or total task times in the tinnitus group, even in trials with neutral stimuli, reinforcing the hypothesis of a generalized effect of tinnitus on cognitive performance [8,11,13,15]. One possible explanation for the generalized depletion of cognitive resources in participants with tinnitus is that they are constantly performing dual tasks, as attention to tinnitus consumes attentional resources [13,15].

Some authors have found that reaction time was longer in the tinnitus group only for the incongruent Stroop trials, and therefore, attention deficits in this population would not be attributed to a generalized depletion of cognitive resources [14]. The dorsal anterior cingulate cortex, activated in Stroop tasks, was recruited in more generalized processing functions, with an influence on brain function as a whole [20]. The auditory modality of the spatial Stroop demonstrated that participants with tinnitus were slower than controls in all stages of the test, regardless of the interference [16]. Our results are consistent with findings of previous studies [8,10,11,13,15,16,20], showing a more widespread effect of tinnitus on Stroop task performance.

There were a few errors in absolute values for the three types of stimuli presented (Table 9), as observed in other studies [8,15,20]. A higher concentration of participants with tinnitus was observed among those who made three or more errors for the three test ranges applied (Table 10).

Previous studies demonstrated no difference in the number of errors between groups with and without tinnitus [8,15]. Our dissonant findings could be explained by our methodology (nonverbal stimuli, free of semantic associations, and did not require more elaborate processing). The Stroop interference conflict can be established in a more basal processing pathway, possibly related to tinnitus.

The WN and NB conditions showed the between-group differences in the distribution of participants regarding error counts (Table 10). In the Study group, there were slightly more participants with noise-like tinnitus than tonal tinnitus (Table 5). It is possible that a larger, more balanced sample, encompassing different tinnitus types, would reveal differences across all stimulus types, as observed for total task time. However, we note this is a speculative observation. This potential relationship warrants dedicated investigation in future trials using larger, subtype-stratified cohorts to determine if stimulus-tinnitus congruency dictates the magnitude of interference.

Another possible explanation for this finding is that most participants in the Study group experienced tinnitus with pitch measured in the 2–8 kHz frequency range (Table 5). Broadband noise stimuli recruit a greater range of auditory nerve fibers, potentially activating regions closer to or within the tinnitus frequency range. In contrast to tonal stimuli, broadband noise encompasses a wider spectrum of frequencies. Sound stimulation using different masking sounds can interfere with the pattern of spontaneous neural firing along the auditory pathway and can temporarily suppress or eliminate tinnitus [39,40]. Sound stimuli compatible with tinnitus in a frequency range may contribute to the magnitude of this phenomenon [40].

In the present study, we applied nonverbal auditory stimuli with distinct physical characteristics. We also included the additional test range (Tinnitus Pitch) to enhance test sensitivity. The analysis, considering the additional test range (Tinnitus Pitch) used with the Study Group, revealed no effect on total task time (Table 11). However, there were more errors in this test track (Table 11 and Table 12). This finding demonstrated that the Tinnitus Pitch stimulus was more sensitive to cognitive interference in the AV-Stroop test. The increased interference in top-down executive control reinforces the idea that the Tinnitus Pitch was more efficient for evaluating participants with tinnitus.

Auditory sensory input disturbance is a condition for tinnitus onset. A deafferented sensory area can result in neuroplastic modifications along and beyond the central auditory pathway [41,42,43,44,45]. The deafferented sensory area presents a specific frequency distribution. The frequencies of tinnitus behavioral expression can be associated with the region of auditory nerve fibers affected. In participants with tinnitus and high-frequency hearing loss, the tinnitus spectrum encompassed the frequencies affected by hearing loss [46]. In the central auditory system, the distribution of nerve fibers follows a defined tonotopic pattern, with modifications in the frequency-specific neural firing pattern at various levels of this pathway [40]. Therefore, the spectral characteristics of the sound stimulus can mobilize specific auditory nerve fibers throughout the auditory pathway. Even in individuals without alterations in conventional audiometry, deafferentation is a condition for the onset of tinnitus, and the spectral characteristics of tinnitus may be related to the deafferented area [40,46].

Our results reinforced this relationship and demonstrated the importance of psychoacoustic measurements for a more in-depth investigation of the symptom, and additionally, as a basis for establishing therapeutic strategies within audiological rehabilitation.

Another possible explanation for the fact that the Tinnitus Pitch test track was more sensitive to demonstrate the interference effect in the Study Group is that such a stimulus would present negative emotional valence, an effect already sought in previous studies that sought to sensitize the test from stimuli related to tinnitus and that involved emotional processing [10,12,18,19]. To investigate this further, future studies should incorporate subjective annoyance ratings for each stimuli track and utilize an emotional Stroop control paradigm.

Furthermore, the Tinnitus Pitch stimulus could activate an important pathway for attentional focus. Tinnitus, as a behaviorally relevant signal, has processing priority at the central nervous system level compared to other competing stimuli [47].

In our study, we found that participants with tinnitus (the Study Group) made more errors in response to WN and NB stimuli compared to the control group. Additionally, a higher percentage of Study group participants made errors on all three stimuli (WN, NB, and TP). While both groups committed errors, only participants in the Study Group exceeded the threshold of three errors per test track (Table 9). Notably, the Tinnitus Pitch test track highlighted the greatest difficulty in attentional and inhibitory control among the Study Group. These findings are shown in Table 11 and Table 12.

Some authors observed the greatest difference in the reaction time rather than in accuracy, stating that tinnitus could affect cognitive efficiency more than cognitive performance [8]. Our findings indicated that tinnitus interference can affect cognitive performance and cognitive efficiency. This suggests that cognitive interference in tinnitus is not necessarily an all-or-nothing failure of accuracy, but rather a persistent drain on processing resources that manifests as slower cognitive throughput.

The cognitive interference observed in our results likely reflects a shared neural substrate between the executive control network and the tinnitus distress network. Regions such as the anterior cingulate cortex and the dorsolateral prefrontal cortex are known to be activated during the resolution of Stroop interference; however, neuroimaging studies consistently show these same areas are chronically recruited in individuals with bothersome tinnitus [35,36]. Our finding of increased errors during pitch-matched tracks suggests a ‘neural bottleneck’ where the shared resources of these cortical areas are overwhelmed by the simultaneous demands of suppressing the internal tinnitus percept and resolving external interference conflict.

The present study highlights a comprehensive and generalized effect of tinnitus on cognitive executive control. Therefore, the audiological approach to tinnitus needs to incorporate measures that assess and help improve attentional control and inhibitory control in individuals with tinnitus. Individuals with tinnitus need to improve their cognitive efficiency and their cognitive performance. This may help to achieve the therapeutic goals of audiological rehabilitation. In addition to the numerous sound-based intervention possibilities, audiological rehabilitation of tinnitus also relies on neurocognitive auditory training.

Unlike another study [17], our findings showed that tinnitus can interfere with cognitive performance, with cognitive failures occurring through the auditory pathway. Our results reinforce alterations in auditory processing in tinnitus [16]. Stroop paradigms involving the auditory modality can be more effective in demonstrating cognitive interference, as previously stated [16].

It is important to note that the error rates observed across all AV-Stroop test tracks remained consistently low. As highlighted in previous literature [8,15], low error frequencies in the Stroop paradigm should be interpreted with caution, as they may suggest that accuracy is not the most sensitive measure for evaluating interference in cognitively intact populations. In our study, the stability of these rates suggests that participants prioritized accuracy, effectively compensating for the conflicting auditory stimuli. Rather than serving as a primary measure of interference, these low error rates confirm that the significant differences found in total task times represent a genuine increase in cognitive processing demand rather than a sacrifice in performance quality.

Additionally, the low frequency of errors observed across all tracks can suggest a ceiling effect in the AV-Stroop task. While the simplicity of the task ensures the test’s clinical accessibility for participants with significant tinnitus distress, it limits the granularity of the error analysis. However, the fact that significant differences were still observed—despite the narrow range of error scores—suggests that the acoustic stimuli compatible with the tinnitus perception (Tinnitus Pitch track) provide a robust enough interference to break through the ceiling effect and reveal differences in inhibitory control.

### 4.4. Limitations and Future Directions

Despite the strengths of this study, certain limitations must be acknowledged. This study does not report test-retest or inter-rater reliability for the AV-Stroop instrument. While the current results establish a foundation for the test’s clinical utility and convergent validity, the stability of the measure over time and across different examiners remains to be quantified. The absence of these metrics is a primary limitation that necessitates future investigation to ensure the instrument’s robustness for long-term clinical monitoring and multi-center research applications.

The presentation software adopted for the AV-Stroop test adaptation did not establish reaction time analysis for the congruent and incongruent trials’ presentations. The study’s objective was not to assess the influence of factors, such as hearing level, education level, depression, anxiety, tinnitus severity, tinnitus characteristics, and symptom onset time, on participants’ performance. While these variables were documented for sample characterization, their specific influence on inhibitory control represents a distinct research question reserved for future multivariate analyses.

We were unable to quantify the ‘rare stimulus effect’ elicited by the 80/20 congruent-to-incongruent ratio. Although this ratio was implemented to increase inhibitory demand—consistent with established oddball paradigms—total task time as the primary outcome measure precludes a separate analysis of trial-specific interference.

The use of a fixed administration order may introduce potential practice or order effects. Although task brevity was designed to minimize cognitive load, the lack of counterbalancing is a limitation to be addressed in future research.

While the MOCA identified a broad range of cognitive performance, it did not correlate with AV-Stroop total task times. This suggests the AV-Stroop may target specific executive resources not captured by global screening tools, though further research is needed to determine the clinical significance of this independence in the tinnitus population.

Tinnitus pitch matching is inherently subjective; therefore, we minimized potential variability by requiring confirmation of the matched frequency. Despite this internal verification, the established limitations of pitch-matching stability in the literature must be considered when interpreting the results.

The observed results yielded significant associations with moderate effect sizes. However, the relatively small sample size and low error variance should be considered when generalizing these findings. Future studies with larger cohorts could further delineate the nuances of AV-Stroop test interference in diverse clinical populations.

Given the exploratory nature of this research, formal corrections for multiple comparisons were not performed. This preserves the power to detect associations in a new assessment paradigm but increases the risk of Type I errors. Accordingly, borderline significant findings (*p*-value approximately 0.05) can be interpreted as preliminary trends that require confirmation in future trials.

The use of a post hoc error threshold (≥3) represents a limitation of the current study. While this cut-off was necessitated by the overall low error rates and the need to characterize specific performance clusters, future studies should utilize larger samples to establish pre-specified, validated clinical benchmarks for AV-Stroop test interference.

We believe that future applications of the AV-Stroop test in tinnitus participants could involve cognitive function assessment and therapeutic monitoring follow-up before and after an intervention. It would be important to investigate functional imaging of the neuronal connectivity network elicited by the AV-Stroop test. It can help clarify the effect of using stimuli compatible with the tinnitus pitch in assessing executive attentional control.

Future studies may clarify these aspects and help guide personalized therapeutic strategies. We agree that enhancing attentional and inhibitory control may be associated with improvements in tinnitus, as suggested by previous studies [14,48].

The audiological rehabilitation process should also consider tinnitus-directed attention. Additionally, the attentional state can be dysfunctional, contributing to the maintenance and chronicity of tinnitus. Neurocognitive auditory training using auditory Stroop tasks [21,22,23] can be a complementary tool in the audiological rehabilitation of tinnitus.

## 5. Conclusions

This study introduced an AV-Stroop test adaptation designed to evaluate executive control in individuals with tinnitus. The results demonstrated that the AV-Stroop test is consistent with the traditional C-Stroop test regarding total task time and error rates, yet it remains independent of mild cognitive impairment screening scores. Participants with tinnitus showed longer total task times and higher error rates compared to controls, suggesting that tinnitus has a broad impact on cognitive performance. Notably, stimuli compatible with the spectral characteristics of the participant’s tinnitus showed more effectiveness in isolating these executive deficits. Clinically, the AV-Stroop provides a specialized metric for the executive interference burden of tinnitus, offering a pathway for future research to investigate how individual factors modulate cognitive interference. This work establishes a foundation for the use of auditory-visual Stroop paradigms in tinnitus auditory interventions.

## Figures and Tables

**Table 1:** Distribution of the test presentation trials.

Trial (#)	Stimulation	Trial (#)	Stimulation	Trial (#)	Stimulation	Trial (#)	Stimulation
1	CONG R	16	CONG R	31	CONG R	46	CONG R
2	CONG L	17	CONG R	32	CONG L	47	CONG L
3	CONG L	18	INCONG R	33	CONG L	48	INCONG L
4	INCONG L	19	CONG R	34	INCONG L	49	CONG R
5	CONG R	20	CONG L	35	CONG R	50	CONG L
6	CONG L	21	CONG L	36	CONG R	51	CONG L
7	CONG L	22	CONG R	37	CONG L	52	CONG R
8	CONG R	23	CONG L	38	INCONG L	53	INCONG R
9	CONG R	24	INCONG L	39	CONG L	54	CONG R
10	INCONG R	25	CONG R	40	CONG R	55	CONG L
11	CONG L	26	CONG L	41	CONG L	56	CONG R
12	CONG R	27	CONG R	42	INCONG L	57	INCONG R
13	CONG L	28	CONG R	43	CONG R	58	CONG L
14	CONG L	29	INCONG R	44	CONG R	59	CONG R
15	CONG L	30	CONG L	45	INCONG R	60	CONG L

Legend: CONG, congruent situation; INCONG, incongruent situation, in relation to the side to which the sound stimuli were presented; L, left; R, right.

**Table 2:** Technical specifications of the AV-Stroop tracks.

Characteristic	Training Track (Habituation)	Test Tracks (WN, NB, PT)
Objective	Procedural fluency, familiarization and habituation	Measurement of cognitive interference
Total Trials	21 trials	61 trials
Congruency Ratio	100% Congruent (after initial binaural)	80% Congruent/20% Incongruent
Feedback	Immediate verbal	No feedback provided

**Table 3:** Eligibility Criteria.

Inclusion Criteria	Age between 18 and 59 years; hearing thresholds within normal standards for conventional audiometry (less than or equal to 20 dBHL at 0.25 to 8 kHz) [24]; education level of 4 or more years; absence of evident and/or diagnosed neurological and/or psychiatric disorders; self-reported visual acuity compatible with the proposed activity.
Exclusion Criteria	Participants who did not sign the informed consent form; family history of hearing loss or external and/or middle ear problems; and a history of ear surgery.

**Table 4:** Comparison between the Study Group (*n* = 30) and the Control Group (*n* = 40) regarding clinical evaluation and questionnaire.

	Study Group (*n* = 30)	Control Group (*n* = 40)	*p*-Value
Age (years)	41.57 (SD 13.51)	41.15 (SD 11.75)	0.704
Gender	Female (*n* = 23)	Female (*n* = 29)	0.693
Hearing Level (0.25–8 kHz RE average, dBHL)	6.27	4.92	0.2
Hearing Level (0.25–8 kHz LE average, dBHL)	6.16	4.68	0.191
Educational Level (years)	4–8 (*n* = 2)	4–8 (*n* = 1)	0.248
	8–12 (*n* = 15)	8–12 (*n* = 14)	
	>12 (*n* = 13)	>12 (*n* = 25)	
HADS-A (score)	0–7 (*n* = 17)	0–7 (*n* = 30)	0.172
	8–11 (*n* = 8)	8–11 (*n* = 8)	
	12–21 (*n* = 5)	12–21 (*n* = 2)	
HADS-D (score)	0–7 (*n* = 24)	0–7 (*n* = 33)	0.144
	8–11 (*n* = 2)	8–11 (*n* = 6)	
	12–21 (*n* = 4)	12–21 (*n* = 1)	

Legend: SD, standard deviation; RE, right ear; LE, left ear; HADS-A, Hospital Anxiety and Depression Scale—Anxiety; HADS-D, Hospital Anxiety and Depression Scale—Depression.

**Table 5:** Characteristics of the Study Group (*n* = 30).

Tinnitus Duration (Months)		49.1 (SD 47.9)	
	**Subtypes**	***n***	**%**
Tinnitus Localization	Bilateral	18	60
Tinnitus Type	Tonal	13	43.3
	Noise-like	17	56.7
Pitch	Low-Frequency (0.25–1 kHz)	5	16.6
	High-Frequency (2–8 kHz)	24	79.8
	WN	1	3.3
Loudness	5–10 dBSL	21	73.3
	15–20 dBSL	6	20
	25 dBSL	2	6.7
THI (score)	Slight (0–16)	10	33.3
	Mild (18–36)	8	26.7
	Moderate (38–56)	7	23.3
	Severe (58–76)	5	16.7

Legend: kHz, kilohertz; WN, white noise; dBSL, decibels sensation level.

**Table 6:** Correlation between the duration of the WN, NB, and PT tests, the duration of the C-Stroop test, and MOCA results.

		WN	NB	PT
Stroop WR	Correlation	0.448	0.576	0.628
	*p*-value	0.002	<0.001	<0.001
	CI (95%)	[0.164; 0.664]	[0.319; 0.754]	[0.386; 0.789]
Stroop CN	Correlation	0.605	0.617	0.690
	*p*-value	<0.001	<0.001	<0.001
	CI (95%)	[0.356; 0.774]	[0.371; 0.782]	[0.470; 0.829]
MOCA	Correlation	−0.184	−0.226	−0.201
	*p*-value	0.225	0.136	0.185
	CI (95%)	[−0.455; 0.118]	[−0.490; 0.076]	[−0.469; 0.101]

Legend: WN, white noise; NB, narrow band; PT, pure tone; MOCA, Montreal Cognitive Assessment; Stroop WR, Stroop word reading; Stroop CN, Stroop color naming; CI, Confidence Interval.

**Table 7:** Correlation between the number of errors in the WN, NB, and PT tests, the number of errors in the C-Stroop test, and MOCA results.

		WN	NB	PT
Stroop WR	Correlation	0.407	0.525	0.392
	*p*-value	0.006	<0.001	0.008
	CI (95%)	[0.116; 0.633]	[0.255; 0.719]	[0.100; 0.622]
Stroop CN	Correlation	0.148	0.371	0.203
	*p*-value	0.331	0.012	0.180
	CI (95%)	[−0.154; 0.425]	[0.077; 0.606]	[−0.099; 0.471]
MOCA	Correlation	−0.221	−0.174	−0.114
	*p*-value	0.144	0.254	0.454
	CI (95%)	[−0.486; 0.081]	[−0.446; 0.129]	[−0.396; 0.186]

Legend: WN, white noise; NB, narrow band; PT, pure tone; MOCA, Montreal Cognitive Assessment; Stroop WR, Stroop word reading; Stroop CN, Stroop color naming; CI, Confidence Interval.

**Table 8:** Comparison of the total task time between the Study and Control Groups in the AV-Stroop test.

AV-Stroop Test’s Track	Total Task Time (s): Mean (SD)		*p*-Value	Effect Size
	Study Group	Control Group		
Training	55.73 (SD 6.64)	52.63 (SD 4.41)	0.008	0.37
WN	140.50 (SD 13.46)	135.40 (SD 7.31)	0.018	0.33
NB	140.70 (SD 12.58)	133.15 (SD 6.62)	0.006	0.38
PT	140.33 (SD 10.48)	132.50 (SD 6.59)	<0.001	0.49

Legend: s, seconds; SD, standard deviation; WN, white noise; NB, narrow band; PT, pure tone.

**Table 9:** Number of errors of the Study and Control Groups on each track of the AV-Stroop test.

# Errors	WN Track Study Group	WN Track Control Group	NB Track Study Group	NB Track Control Group	PT Track Study Group	PT Track Control Group
Mean	1.50	0.80	1.87	0.90	1.58	0.78
Median	1.00	1	1.00	1	1.00	1
SD	1.76	0.87	2.24	0.83	1.98	0.90
Minimum	0	0	0	0	0	0
Maximum	9	3	10	3	9	3

Legend: SD, standard deviation; WN, white noise; NB, narrow band; PT, pure tone.

**Table 10:** Comparison of participants’ distribution of the Study and Control Groups based on error frequency thresholds (<3 vs. >3 errors) in the AV-Stroop test. *p*-values indicate the results of comparing the categorical frequency between the Study and Control groups for each specific AV-Stroop track (WN, NB, and PT).

AV-Stroop Test Track	# Errors	Participants (*n*, %)		*p*-Value
		Study Group	Control Group	
WN	<3 Errors	22 (73.3)	39 (97.5)	0.045
	≥3 Errors	8 (26.7)	1 (2.5)	
NB	<3 Errors	21 (70)	39 (97.5)	0.012
	≥3 Errors	9 (30)	1 (2.5)	
PT	<3 Errors	23 (76.7)	37 (92.5)	0.079
	≥3 Errors	7 (23.3)	3 (7.5)	

Legend: WN, white noise; NB, narrow band; PT, pure tone; *n*, number of participants; %, percentage.

**Table 11:** Within-group comparison of the total task time and the number of errors in the Study Group across standard test tracks (WN, NB, PT) and the personalized Tinnitus Pitch track (customized via pitch-matching assessment). *p*-values represent the results of the Friedman Test; effect size is reported as Kendall’s W.

	AV-Stroop Test Track	Results	*p*-Value	Effect Size
Total task time (s)	WN	140.5 (SD 13.5)	0.907	
	NB	140.7 (SD 12.6)		
	PT	140.3 (SD 10.5)		
	Tinnitus Pitch	141.7 (SD 15.8)		
Errors (*n*)	WN	1.5 (SD 1.5)	0.020	0.11
	NB	1.9 (SD 2.5)		
	PT	1.6 (SD 2.1)		
	Tinnitus Pitch	2.3 (SD 2.5)		

Legend: s, seconds; SD, standard deviation; WN, white noise; NB, narrow band; PT, pure tone; *n*, number.

**Table 12:** Comparison of errors across different test tracks within the Study Group.

Multiple Comparisons (Errors)	*p*-Value	Effect Size
WN vs. NB	0.617	
WN vs. PT	0.439	
WN vs. Tinnitus Pitch	0.059	
NB vs. PT	0.439	
NB vs. Tinnitus Pitch	0.046	0.07
PT vs. Tinnitus Pitch	0.003	0.15

Legend: WN, white noise; NB, narrow band; PT, pure tone; vs., versus.

## Data Availability

The datasets generated and analyzed during the current study are publicly available in the Universidade Federal de São Paulo Repository at https://repositorio.unifesp.br/items/be83cdc6-cba0-4de7-926e-b422352ea8a2 (accessed on 15 January 2026).

## Abbreviations

The following abbreviations are used in this manuscript:

AV-Stroop Test	Auditory-Visual Stroop Test
C-Stroop Test	Conventional Stroop Test
HADS	Hospital Anxiety and Depression Scale
MOCA	Montreal Cognitive Assessment
WN	White Noise
PT	Pure Tone
NB	Narrow Band
